# Pore-scale numerical simulation of *in situ* microemulsion formation and enhanced oil recovery in porous media

**DOI:** 10.3389/fchem.2025.1601086

**Published:** 2025-05-19

**Authors:** Yingxue Hu, Kai Dong, Dan Zhang, Tianjiang Wu, Wei Xu, Zhaolin Gu

**Affiliations:** ^1^ School of Human Settlements and Civil Engineering, Xi’an Jiaotong University, Xi’an, China; ^2^ Key Laboratory of Synthetic and Natural Functional Molecule Chemistry of Ministry of Education, College of Chemistry and Materials Science, Northwest University, Xi’an, China; ^3^ Oil and Gas Technology Research Institute of Changqing Oilfield, China National Petroleum Corporation, Xi’an, China

**Keywords:** *in situ* microemulsion, porous media, solubilization, viscosity modification, porescale

## Abstract

*In situ* microemulsion has emerged as an advanced tertiary oil recovery technique that utilizes the injection of surfactant solutions to improve displacement efficiency through spontaneous microemulsification. This study presents a novel pore-scale numerical model to simulate the dynamic process of *in situ* microemulsion formation during surfactant-cosolvent-salt flooding in complex porous media. Through comprehensive numerical simulations based on realistic rock geometries, we systematically investigated the spatiotemporal evolution of phase distributions and identified critical mechanisms governing oil mobilization. The developed model incorporates four fundamental characteristics of microemulsion systems: interfacial tension reduction, viscosity modification, wettability alteration, and enhanced solubilization capacity. During the microemulsion-forming surfactant flooding in a realistic rock medium, the *in situ* formed microemulsion was observed at the interface between oil and aqueous. The *in situ* microemulsion flooding can significantly improve the recovery rate under the combined effect of multiple factors. Increasing the viscosity of the *in situ* formed microemulsion can enhance the oil recovery during the microemulsion-forming surfactant flooding in the complex porous media. Under water-wet conditions, the oil-water interface stays at the junction of the throat and the pore space, which contributes to the formation of microemulsions and thus to the enhancement of recovery. This study provides a better understanding of the *in situ* microemulsion formation and the mechanisms of enhanced oil recovery in complex porous media.

## 1 Introduction

Tertiary oil recovery, particularly chemical flooding, has emerged as an essential strategy to enhance oil recovery from residual and remaining oil post-water flooding (Park et al., 2021; Tavakkoli et al., 2022). Among chemical flooding techniques, microemulsion flooding has gained significant attention due to its unique ability to reduce interfacial tension (IFT), alter wettability, and mobilize trapped residual oil in porous media (Qin et al., 2020; Das et al., 2021; Wu et al., 2024; Koreh et al., 2025). Microemulsions, thermodynamically stable dispersions of surfactant, cosolvent, salt, and oil, have demonstrated remarkable success in improving oil recovery.

Microemulsions are classified into three types based on their phase behavior: Winsor I (oil-in-water, O/W), Winsor II (water-in-oil, W/O), and Winsor III (bicontinuous). Winsor I microemulsions consist of nano-scale oil droplets dispersed in a continuous water phase, stabilized by surfactant molecules at the oil-water interface. Winsor II microemulsions feature water droplets dispersed in a continuous oil phase, while Winsor III microemulsions exhibit a bicontinuous structure where both oil and water phases are interconnected, forming a sponge-like network (Scriven, 1976; Zhao et al., 2025). The formation of these microemulsions is highly dependent on surfactant concentration, cosurfactant selection, salinity, and oil-to-water ratio (Budhathoki et al., 2016). Salinity and oil-water ratio scanning experiments are critical for identifying optimal conditions for microemulsion formation, ensuring maximum oil recovery efficiency (Zhou et al., 2020; Mariyate and Bera, 2022).

The unique physical properties of microemulsions, such as solubilization capacity, ultra-low IFT, wettability alteration, and viscosity modulation, collectively contribute to enhanced oil recovery (EOR). Solubilization enables microemulsions to dissolve crude oil and water, improving oil mobility (Wei et al., 2011). Ultra-low IFT reduces the oil-water interfacial energy, facilitating the detachment of residual oil (Das et al., 2021; Li et al., 2025). Wettability alteration adjusts the wetting state of rock surfaces, optimizing oil-water flow paths, while viscosity modulation enhances the sweep efficiency of displacing fluids (Yang et al., 2021). These properties make microemulsions highly effective in EOR applications.

Two primary methods are employed to utilize microemulsions for EOR: *ex-situ* and *in situ*. The *ex-situ* method involves injecting pre-prepared microemulsions into the reservoir (Zhou et al., 2020). While this approach has demonstrated significant oil recovery enhancement, it faces challenges such as high injection pressures and potential incompatibility with reservoir conditions (Zhu et al., 2022). In contrast, the *in situ* method involves injecting a microemulsion-forming surfactant system into the formation, where microemulsions form spontaneously within the pores (Mo et al., 2023). This method leverages the natural conditions of the reservoir, offering better adaptability and cost-effectiveness. However, the pore-scale mechanisms governing microemulsion-assisted oil recovery remain poorly understood, limiting the optimization of this technology for field applications.

Advanced visualization techniques, including X-ray computed tomography (Hu et al., 2020), nuclear magnetic resonance (Govindarajan et al., 2020), and microfluidics (Fu et al., 2016), have facilitated the analysis of microemulsion formation and EOR in complex porous media. These technologies provide high-precision experimental data, revealing the formation mechanisms, phase behavior, and oil displacement efficiency of microemulsions. For instance, Ott et al. (2020) employed micro-CT to investigate the *in situ* emulsification behavior of surfactants and crude oil during alkali flooding, providing critical insights into the changes in interfacial tension and oil-water phase distribution. Unsal et al. (2019) utilized synchrotron-based X-ray micro-tomography to study the dynamic changes in emulsification under optimal salinity conditions, highlighting the critical role of salinity in emulsification efficiency Alzahid et al. (2019) systematically investigated the pore-scale flow characteristics of surfactant flooding under varying salinity conditions, demonstrating the significant impact of salinity on emulsified phase properties and oil-water distribution. She et al. (2021) further advanced the field by employing three-dimensional visualization techniques to study the role of *in situ* emulsification in alkali flooding, revealing the formation, migration, and trapping mechanisms of emulsions in porous media.

Despite these advancements, visualizing the dynamic distribution of microemulsion concentrations within pores remains challenging due to the temporal and spatial limitations of X-ray CT. Two-dimensional micromodels with high-speed cameras have been employed to capture near real-time emulsification dynamics. For example, Unsal et al. (2016) used microfluidic flow experiments to study the *in situ* formation of microemulsions by co-injecting *n*-decane and surfactant solutions into a T-junction capillary geometry. Broens and Unsal (2018) investigated emulsification kinetics in quasi-miscible flow conditions, while Tagavifar et al. (2017) analyzed phase formation and spatial configurations in a 2.5-dimensional (2.5D) micromodel. Zhao et al. (2022) demonstrated *in situ* emulsification using on-chip experiments, and Xu et al. (2024) studied the formation and transport mechanisms of microemulsions in fractured porous micromodels. Nevertheless, current visualization techniques remain inadequate for accurately quantifying microemulsion concentrations within complex pore structures, thereby constraining the comprehensive understanding of microemulsion formation and enhanced oil recovery (EOR) mechanisms. Pore-scale numerical simulations have emerged as a promising approach for investigating oil-water interface dynamics in porous media during chemical flooding processes, including surfactant, polymer, and nanoparticle flooding. However, microemulsion flooding presents additional complexities, as it involves not only multiphase flow but also requires consideration of multiphase mass transfer processes within intricate pore networks that has received limited research attention to date.

To overcome these research gaps, the present study develops a novel pore-scale simulation framework to systematically examine microemulsion formation and EOR mechanisms in porous media. By analyzing microemulsion behavior in a simplified T-junction structure, we aim to elucidate the roles of IFT reduction, wettability alteration, viscosity enhancement, and solubilization in oil displacement. Furthermore, a real complex porous medium is reconstructed from digital rock, and the pore-scale dynamics of the emulsification process are coupled with fluid flow. This study leverages advanced imaging techniques and numerical simulations to provide a comprehensive pore-scale investigation of microemulsion-enhanced oil recovery, bridging the gap between laboratory-scale observations and field-scale applications. The findings are expected to contribute to the development of more effective EOR strategies, ultimately improving oil recovery efficiency and reducing the environmental footprint of hydrocarbon extraction.

## 2 Numerical method

This study employs the conventional Volume of Fluids (VoF) method to simulate two-phase displacement within porous media and accurately track the interface between water and oil. In the VoF method, an indicator function, denoted as α, is defined throughout the flow domain, representing the volume fraction of one of the fluids within each computational grid cell. Specifically, *α* = 1 indicates that a cell is entirely occupied by water, while *α* = 0 signifies that the cell is completely filled with oil. The indicator function serves as a critical tool for identifying and tracking the interface between the two phases. The governing equations for the system, which describe the behavior of two incompressible, isothermal, and immiscible fluids (such as water and oil), are presented as follows:

$$\frac{\partial \rho u}{\partial t}+\nabla \cdot \left(\rho \text{uu}\right)-\nabla \cdot \left(\mu \left(\nabla u+{\left(\nabla u\right)}^{T}\right)\right)=-\nabla p+F_{\sigma }$$ (1)

∇·u=0 (2)

$$\rho =\alpha \rho_{w}+\left(1-\alpha \right)\rho_{o}$$ (3)

$$\mu =\alpha \mu_{w}+\left(1-\alpha \right)\mu_{o}$$ (4)

In the above equations, Equations 1, 2 are momentum and continuity equations of the fluid, where *ρ*, ***u***, p, and ***F***_σ_ are density, fluid velocity, dynamic pressure, and surface tension, respectively. *t* is the time. The effect of gravity is ignored in two-dimensional models. *α* denotes the volume fraction of the water phase and correspondingly (1−*α*) the oil phase in Equations 3, 4.

The surface tension ***F***_σ_ in Equation 2 is modeled as continuum surface force (CSF) and calculated by

$$F_{\sigma }=\sigma \kappa \nabla \alpha$$ (5)

where *σ* is the surface tension coefficient, and *κ* is the curvature of the water-oil interface in Equation 5, which can be approximated as follows

κ=−∇·n (6)

**n** is the interface normal unit vector (in Equations 6-8) given by

$$n=\frac{\nabla \alpha }{\left|\nabla \alpha \right|}$$ (7)

To accurately model the wettability of the porous media, the contact angle is imposed as a wetting boundary condition. The normal unit vector of the interface at the wall boundary is adjusted according to the following equation:

$$n=n_{p}\cos\theta +n_{t}\sin\theta$$ (8)

where **n**_*p*_ is the unit vector perpendicular to the wall, **n**_*t*_ is the unit vector tangential to the wall, and *θ* is the contact angle. In this way, the interface can form the prescribed angle *θ* when in contact with the wall.

In this study, we will only focus on the Winsor I (oil in water, O/W) microemulsion. The surfactant system forming the microemulsion is an aqueous solute and the microemulsion formed is treated as a solute in water.

To track the concentration of microemulsion-forming surfactant system in water, the mass conservation equation for the surfactant is calculated:

$$\frac{\partial \alpha C}{\partial t}+\nabla \cdot \left(C\alpha u\right)=\nabla \cdot \left({\alpha D}_{c}\nabla C\right)-S_{c}$$ (9)

where *α* is 1, representing surfactant in water phase, *C* and *D*_c_ are the concentration and diffusion coefficient of surfactant in water, respectively. The source term *S*_c_ denotes the rate of consumption of surfactant in Equation 9.

To track the concentration of microemulsion in water, the mass conservation equation for the microemulsion is calculated:

$$\frac{\partial \alpha C_{e}}{\partial t}+\nabla \cdot \left(\alpha C_{e}u\right)-\nabla \cdot \left(\alpha D_{e}{\nabla C}_{e}\right)=S_{e}$$ (10)

where *α* is 1, representing microemulsion in water phase, *C*_*e*_ and *D*_e_ are the concentration and diffusion coefficient of microemulsion in water, respectively. The source term *S*_e_ denotes the rate of production of microemulsion in Equation 10.

The evolution of water or oil phase is governed by the mass balance equation:

$$\frac{\partial \alpha }{\partial t}+\nabla \cdot \left(\alpha u\right)=S_{\alpha }$$ (11)

The source term is calculated by:

$$S_{a}=-k_{\alpha }C\left(C_{e}-C_{e\infty }\right)\delta$$ (12)

where *k*_α_ and *δ* are the rate of emulsification, and specific interfacial area between oil and water in Equations 11, 12.

The mathematical model has been implemented within the open-source computational fluid dynamics (CFD) platform OpenFOAM. The governing equations are discretized using a finite-volume method and solved sequentially. The pressure–velocity coupling is addressed through a predictor–corrector approach, leveraging the Pressure-Implicit with Splitting of Operators (PISO) algorithm to ensure numerical stability and accuracy.

## 3 Results and discussion

### 3.1 Modelling the unique physical properties of microemulsions

Microemulsions exhibit several unique physical properties that contribute to enhanced oil recovery. The core mechanism involves the formation of nanoscale micellar systems facilitated by surfactants, which lead to viscosity enhancement, interfacial tension reduction, solubilization, and wettability alteration. These properties often interact synergistically during the oil displacement process rather than acting independently. However, to validate the capability of numerical simulations in capturing these properties and to analyze the individual contribution of each factor to oil recovery, this section examines each physical property separately.

#### 3.1.1 Viscosity enhancement

To investigate the effect of viscosity enhancement, water and a microemulsion-forming surfactant were injected into a capillary tube initially saturated with oil. The flow rates of both fluids were maintained at the same level. In this scenario, the effects of interfacial tension reduction, solubilization, and wettability alteration were intentionally excluded from consideration. Notably, the viscosity of the microemulsion-forming surfactant system is comparable to that of water. Therefore, any observed differences in oil-water displacement within the capillary tube can be attributed solely to the viscosity enhancement resulting from the *in situ* formation of microemulsions.

**FIGURE 1:**
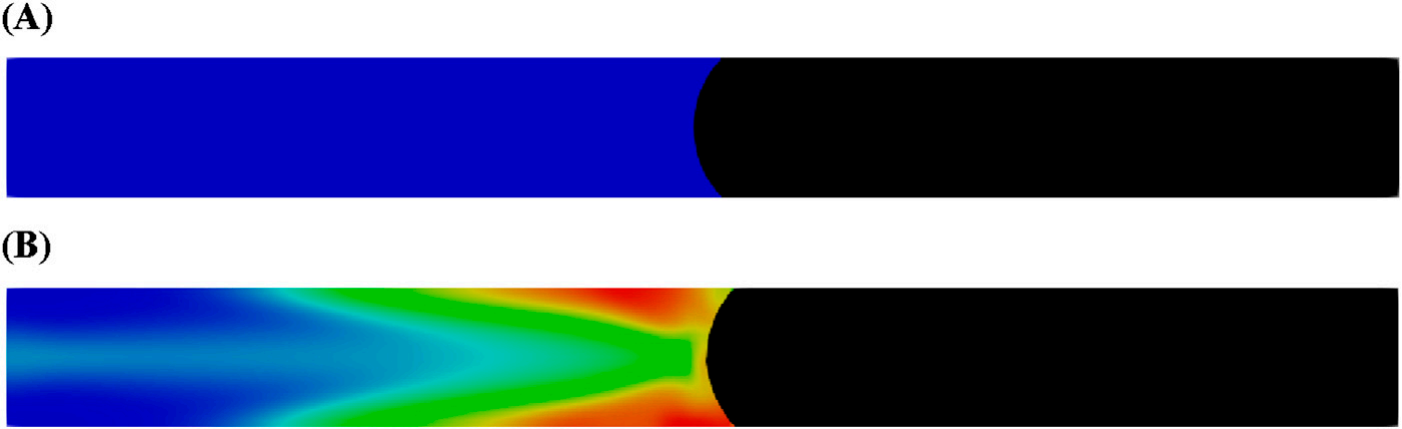
Oil and aqueous distribution in capillary tube after injection of **(A)** water and **(B)** microemulsion-forming surfactant solution. Considering the viscosity enhancement effect of microemulsion during the two-phase displacement process. Black and blue represents oil and water, respectively.

Figure 1 illustrates the distribution of oil and aqueous in the capillary tube following the injection of water and the microemulsion-forming surfactant. A two-dimensional capillary tube with a diameter of 1 mm and a length of 10 mm was used in this study. As depicted in Figure 1B, the injection of the surfactant led to the *in situ* formation of microemulsions near the oil-water interface. The red regions in the figure indicate a high concentration of microemulsion within the aqueous phase. The microemulsion accumulates along the walls of the capillary tube due to the higher flow velocity in the center of the tube. Although no significant differences are observed at the oil-water interface between the two injection fluids, the flow resistance during injection differs due to the viscosity-enhancing effect of the microemulsion.

**FIGURE 2:**
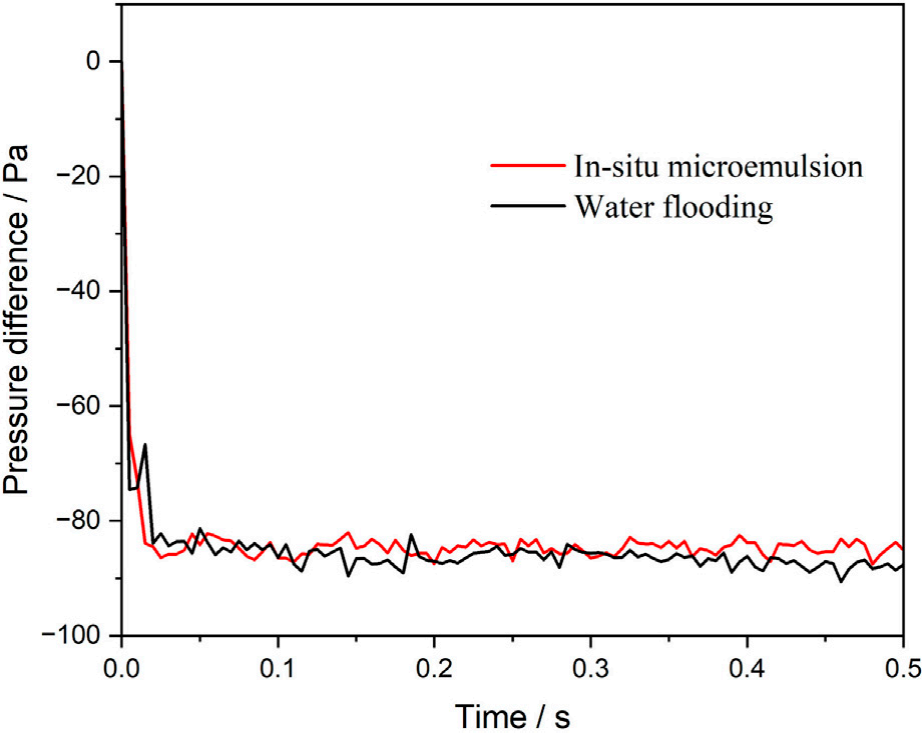
Pressure difference between the inlet and the outlet during water and surfactant microemulsion-forming surfactant flooding. Considering the viscosity enhancement effect of microemulsion.

Figure 2 presents the pressure difference between the inlet and outlet during the injection of water and the microemulsion-forming surfactant. The negative pressure difference values indicate that the flooding process is driven by spontaneous imbibition, a consequence of the strongly water-wet conditions. Initially, the absolute value of the pressure difference increases sharply as a meniscus form at the oil-water interface. Subsequently, as the injection continues, the pressure difference between the inlet and outlet increases at a slower rate. Overall, after the establishment of the interface, the surfactant-driven flooding exhibits a lower pressure difference compared to water flooding. This difference arises from the *in situ* formation of a more viscous microemulsion during surfactant flooding. While this increased viscosity imposes greater flow resistance within the capillary tube, it can lead to improved oil recovery in actual reservoir conditions.

#### 3.1.2 Interfacial tension reduction

**FIGURE 3:**
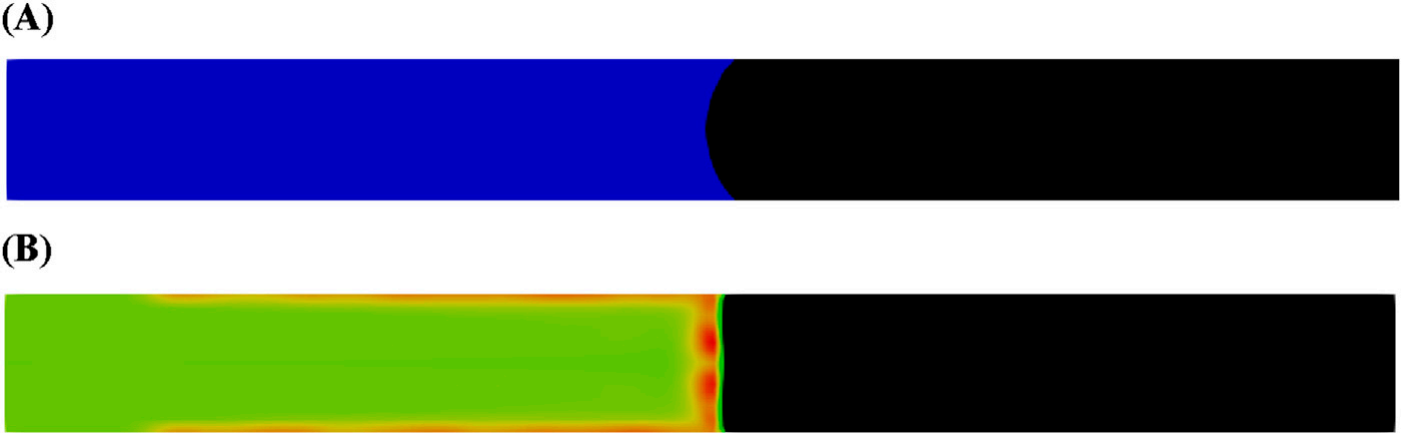
Oil and aqueous distribution in capillary tube after injection of **(A)** water and **(B)** microemulsion-forming surfactant solution. Considering the interfacial tension reduction effect of microemulsion during the two-phase displacement process. Black and blue represents oil and water, respectively.

To examine the effect of interfacial tension reduction on oil-water displacement in porous media, the interfacial tension values between oil and water and between oil and microemulsion were set to 50 mN/m and 0.01 mN/m, respectively. Figure 3 compares the distribution of oil and aqueous in the capillary tube after the injection of water and the microemulsion-forming surfactant. The results demonstrate that the interfacial tension of the microemulsion significantly influences the meniscus formation at the oil-water interface. Specifically, the thickness of the *in situ*-formed microemulsion layer with ultra-low interfacial tension is considerably smaller than that observed under high interfacial tension conditions (Figure 1B). In the capillary tube, capillary forces drive the oil-water displacement process. According to the Young–Laplace equation, higher interfacial tension enhances capillary forces, leading to increased imbibition rates. However, the ultra-low interfacial tension associated with microemulsions alters this dynamic, reducing the capillary forces and thereby affecting the displacement process.

**FIGURE 4:**
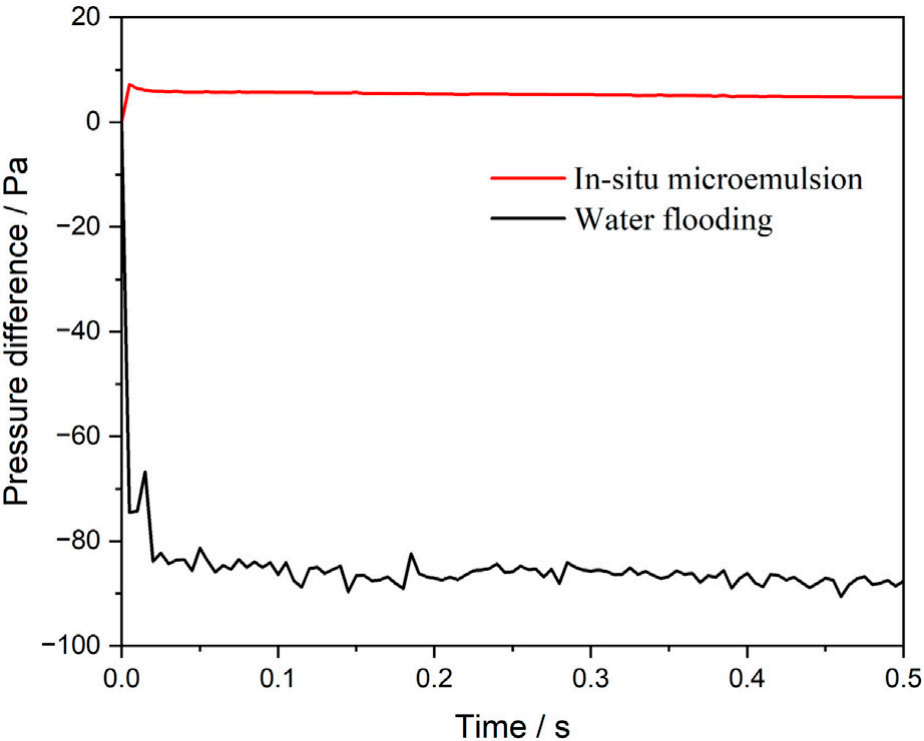
Pressure difference between the inlet and the outlet during water and microemulsion-forming surfactant flooding. Considering the interfacial tension reduction effect of microemulsion.

Figure 4 illustrates the pressure difference between the inlet and outlet during the injection of water and the microemulsion-forming surfactant, considering the effect of interfacial tension reduction. Notably, the pressure difference exhibits contrasting behaviors for the two flooding processes: it is negative for water flooding but positive for microemulsion-forming surfactant flooding. This indicates that capillary forces act as a driving force during water flooding but as a resistance force during microemulsion-forming surfactant flooding. As expected, the magnitude of the pressure difference during microemulsion-forming surfactant flooding is significantly smaller, approximately 5.2 Pa, compared to 86 Pa during water flooding. For microemulsion-forming surfactant flooding, the pressure difference is primarily attributed to flow resistance, with capillary forces playing a minor role.

#### 3.1.3 Solubilization

The solubilization effect plays a critical role in microemulsion-forming surfactant flooding, particularly in recovering oil from dead-end pores. While reducing interfacial tension is often ineffective for mobilizing residual oil in such regions, the solubilization capability of microemulsions significantly enhances the recovery of trapped oil. To investigate how the solubilization effect of *in situ*-formed microemulsions improves the mobilization of residual oil in dead-end pores, a comparative study was conducted using water flooding and microemulsion-forming surfactant flooding in a T-junction model. The capillary tube (1 mm diameter × 3 mm length) connects to a dead-end pore (1 mm × 1 mm square).

**FIGURE 5:**
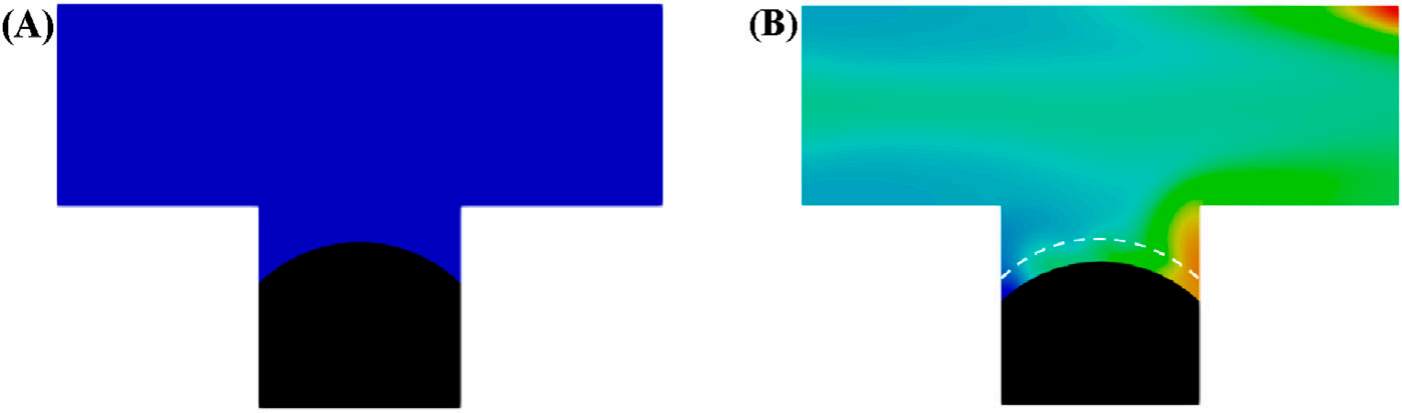
Oil and aqueous distribution in capillary tube after injection of **(A)** water and **(B)** microemulsion-forming surfactant solution. Considering the solubilization effect of microemulsion during the two-phase displacement process. Black and blue represents oil and water, respectively.

As illustrated in Figure 5A, a residual oil droplet forms in the dead-end pore during water flooding. Once the droplet is trapped, continued water injection fails to mobilize it further. In contrast, during microemulsion-forming surfactant flooding, where solubilization effects are considered, a residual oil droplet initially forms in the dead-end pore as well. However, as shown in Figure 5B, the oil-water interface, initially positioned at the dashed line, gradually recedes as the microemulsion-forming surfactant is continuously injected. This receding interface indicates that the residual oil is progressively solubilized into the aqueous phase. With sufficient injection time, the residual oil in the dead-end pore can be entirely recovered.

**FIGURE 6:**
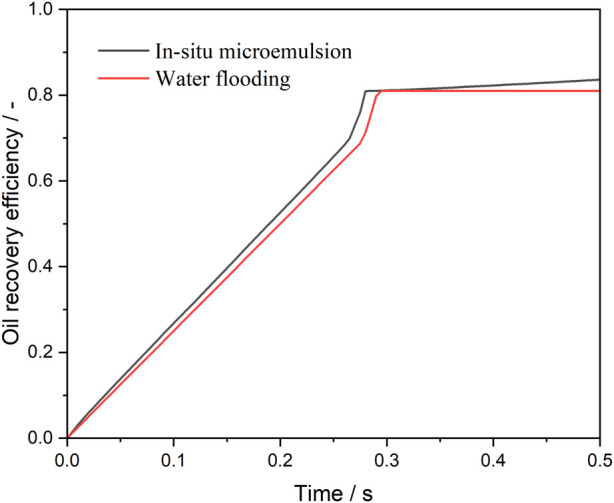
Oil recovery efficiency during water and microemulsion-forming surfactant flooding. Considering the solubilization effect of microemulsion.

Figure 6 presents the oil recovery efficiency profiles during water and microemulsion-forming surfactant injection, highlighting the solubilization effect. Before breakthrough, the oil saturation within the pore space decreases linearly as water or microemulsion-forming surfactant is injected. At the pinch-off stage, the continuous oil phase within the pore is divided into two parts: one portion becomes trapped in the pore, while the other is displaced out of the pore. This process occurs rapidly, resulting in a sharp decline in oil saturation, as depicted in Figure 6. After breakthrough, the oil saturation during water flooding stabilizes, indicating no further recovery. In contrast, during microemulsion-forming surfactant flooding, the oil saturation continues to decrease gradually due to the solubilization effect. Consequently, the ultimate oil recovery efficiency of microemulsion-forming surfactant flooding is significantly higher than that of water flooding, a result attributed to the solubilization effect of *in situ*-formed microemulsions.

#### 3.1.4 Wettability alternation

**FIGURE 7:**
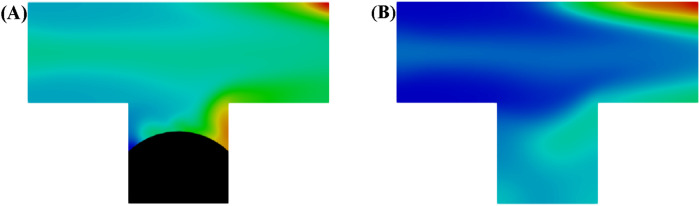
Oil and aqueous distribution in capillary tube after injection of **(A)** water and **(B)** microemulsion-forming surfactant solution. Considering the wettability alternation effect of microemulsion during the two-phase displacement process. Black and blue represents oil and water, respectively.

In microemulsions, surfactants adsorb onto rock surfaces, shifting their wettability from oil-wet to water-wet. This allows the aqueous phase to penetrate pores more effectively, detach oil films, and carry the residual oil out. In order to investigate how *in situ* formation of microemulsions provides recovery through wettability alternation, two kinds of wall wettability were set with contact angles of 45° and 15°, respectively. Figure 7 shows the dynamics distribution of oil and aqueous phase in the T-junction model. Cloud diagram showing the concentration distribution of microemulsion in water at steady-state. At contact angle of 45°, a residual oil droplet was formed within the pore space after microemulsion-forming surfactant flooding, whereas when the contact angle was alternated to 15°, all oil phases in the channel were expelled out.

**FIGURE 8:**
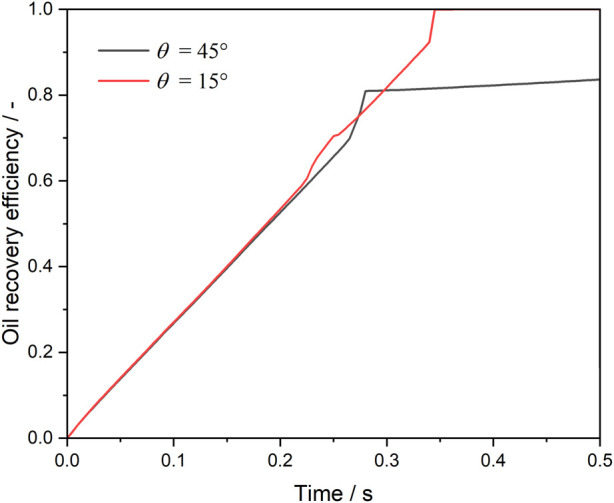
Oil recovery efficiency during water and microemulsion-forming surfactant flooding. Considering the wettability alternation effect of microemulsion.

Figure 8 shows the oil recovery efficiency during water and microemulsion-forming surfactant injection by considering the wettability alternation effect. As expected, before the breakthrough, the oil saturation within the pore space decreases linearly as water or microemulsion-forming surfactant is injected into the pore space. The ultimate oil saturation with contact angles of 45° and 15° are 17% and 0%, respectively. The results indicate that the stronger the water-wet strength, the less residual oil in the dead-end pore.

### 3.2 Oil and water distribution during microemulsion-forming surfactant flooding

To investigate the behavior of *in situ* microemulsion formation in complex porous structures, direct numerical simulations of microemulsion-forming surfactant flooding were conducted in a porous medium reconstructed from a digital rock. The size of the porous medium is 3 mm × 3 mm and corresponding the mean pores diameter is approximately 100 μm. The simulation accounts for the unique properties of microemulsions, including viscosity enhancement, interfacial tension reduction, solubilization, and wettability alteration. The porous medium was initially saturated with oil, and microemulsion-forming surfactant was injected from the left inlet, displacing oil toward the right outlet.

**FIGURE 9:**
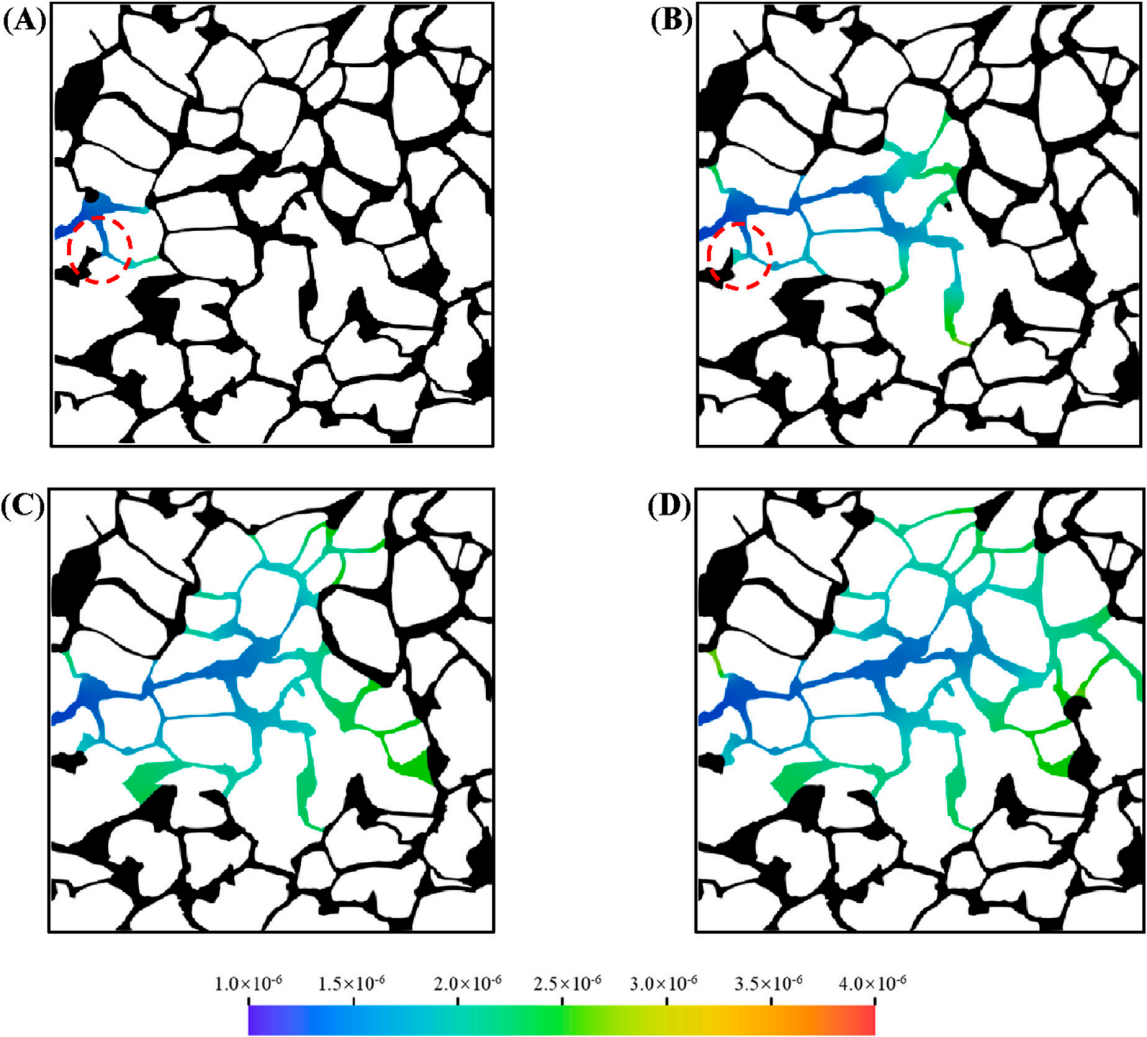
Oil and water distribution in porous media during microemulsion-forming surfactant flooding at different: **(A)** 0.1 s, **(B)** 0.4 s, **(C)** 0.8 s and **(D)** 1.2 s.

Figure 9 illustrates the distribution of oil and aqueous phases in the porous medium at different times, where black represents the oil phase and colors represent the aqueous phase. At the early stage, a low concentration of microemulsion forms near the oil-water interface, while no microemulsion is observed near the inlet, as shown in Figure 9A. As the injection of the microemulsion-forming surfactant solution continues, the concentration of microemulsion in the aqueous phase increases. By *t* = 0.4 s, a mainstream channel with a lower microemulsion concentration emerges in the porous medium, while higher microemulsion concentrations are observed in the elongated pore throats on either side (Figure 9A). The flow stagnation zones in the pore throats restrict the movement of microemulsion, which can only enter the mainstream channel through diffusion. Due to the combined effects of viscosity enhancement and interfacial tension reduction associated with the *in situ* formed microemulsion, the swept area within the porous medium gradually expands, as depicted in Figures 9C,D.

During microemulsion-forming surfactant flooding, flow-advantaged channels with low microemulsion concentrations persist within the porous medium. Similar to conventional water flooding, a portion of the residual oil remains trapped in the pore space. However, the dynamic process of residual oil mobilization during *in situ* microemulsion formation is highlighted by two selected residual oil droplets (marked by red circles in Figure 9). In Region-1, the interface of the residual oil droplet recedes continuously, and its size diminishes over time, accompanied by a gradual increase in microemulsion concentration in the pore throat. In Region-2, smaller residual oil droplets are rapidly and completely dissolved, demonstrating the solubilization effect of the microemulsion.

**FIGURE 10:**
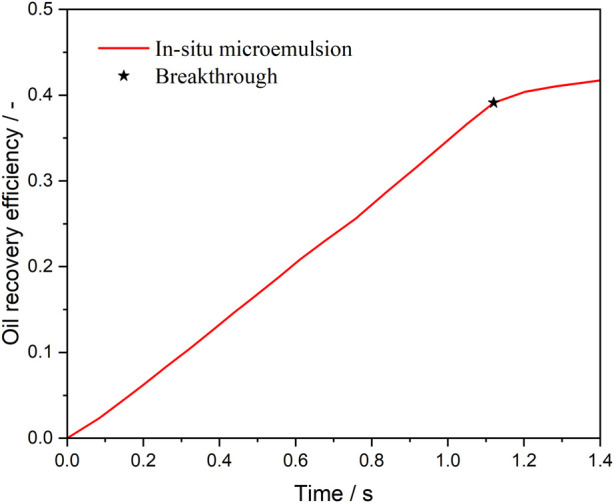
Oil recovery efficiency during microemulsion-forming surfactant flooding.

Figure 10 presents the oil recovery efficiency profiles during microemulsion-forming surfactant flooding in the complex porous medium. Breakthrough refers to the moment when the displacing fluid (microemulsion-forming surfactant) first arrives at the outlet of the porous media. Before breakthrough, the oil saturation decreases linearly as water or microemulsion-forming surfactant is injected. Notably, the rate of oil saturation reduction is faster during microemulsion-forming surfactant flooding due to the solubilization effect of the microemulsion. After breakthrough, the oil saturation during water flooding stabilizes, while it continues to decrease slowly during microemulsion-forming surfactant flooding. This gradual reduction in oil saturation is attributed to the diminishing solubilization effect as the formed microemulsion aggregates at the oil-water interface. As shown in Figure 10, the rate of Oil recovery efficiency increase slows over time in the second stage. Additionally, as the remaining oil saturation decreases, the oil-water interface area—the primary site of *in situ* microemulsion formation—also diminishes, further limiting the solubilization process.

### 3.3 Effect of viscosity on microemulsion-forming surfactant flooding

In batch experiments, microemulsions with varying physical properties can be formulated by adjusting the composition of different surfactants. One of the most critical parameters influencing oil recovery is the viscosity of the *in situ*-formed microemulsion. To investigate the effect of microemulsion viscosity on oil recovery in porous media, three simulation cases were conducted with different viscosity ratios. The viscosity ratio, defined as *M* = *μ*_e_/*μ*_w_, where *μ*_e_ and *μ*_w_ represent the viscosities of the microemulsion and water, respectively, was used to characterize the viscosity-enhancing effect of the microemulsion.

**FIGURE 11:**
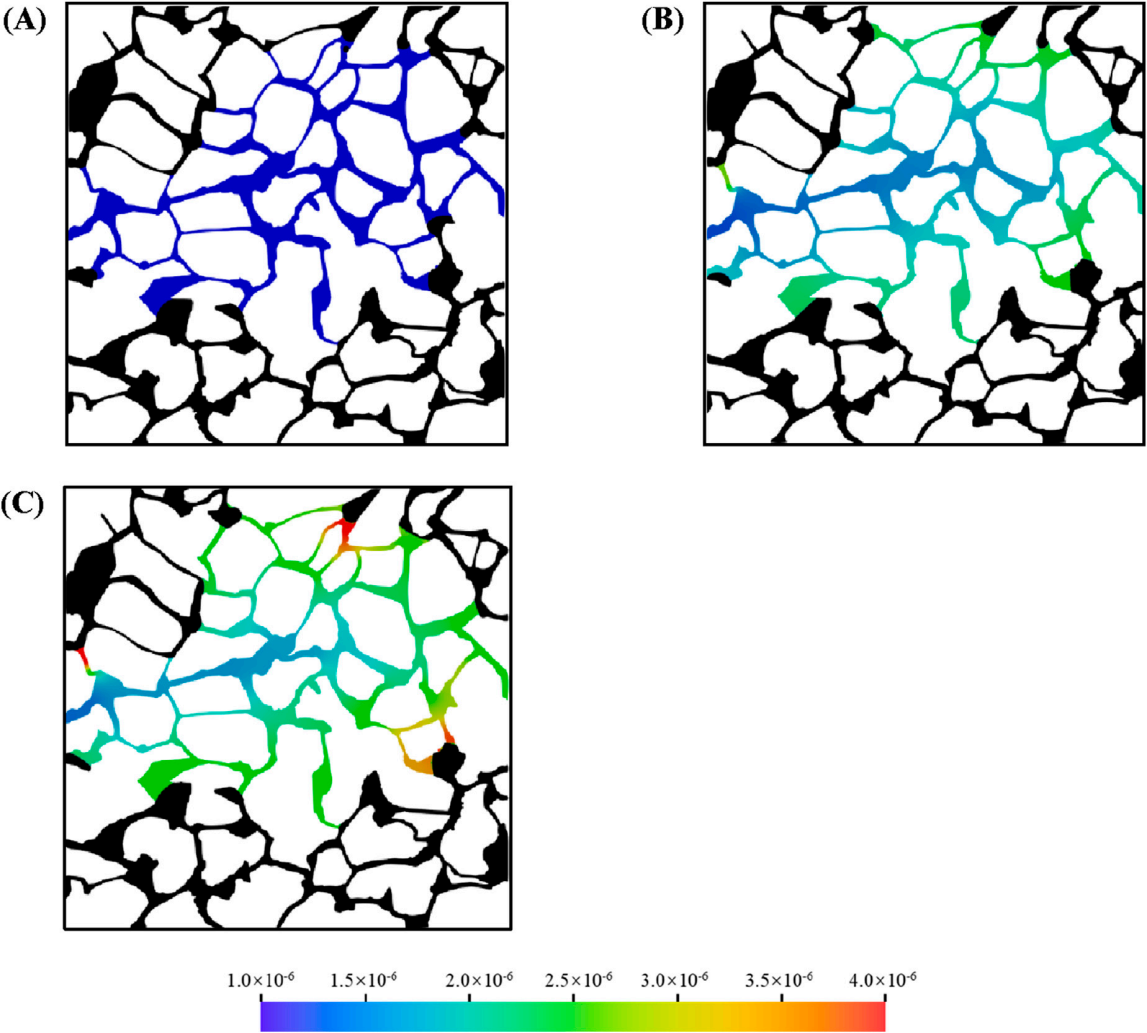
Oil and aqueous distribution in porous media during microemulsion-forming surfactant flooding at different: **(A)** 1 time, **(B)** 3 times and **(C)** 4 times.

Figure 11 illustrates the oil recovery efficiency profiles during microemulsion-forming surfactant flooding in a complex porous medium for viscosity ratios of *M* = 1, *M* = 3, and *M* = 4. The color gradient represents the viscosity distribution of the aqueous phase. As shown in Figure 11A, when *M* = 1, the microemulsion formed *in situ* at the oil-water interface exhibits a viscosity identical to that of water, resulting in a uniform viscosity distribution across the aqueous phase. In contrast, when the viscosity-enhancing effect of the microemulsion is considered *M* = 3 and *M* = 4), the viscosity of the aqueous phase becomes non-uniform. Specifically, the viscosity is lower in the main flow channels, where microemulsion concentration is reduced, and higher at the oil-water interface, where microemulsion concentration is elevated. As the viscosity of the *in situ*-formed microemulsion increases, the oil in regions 1, 2, 3, and 4 is progressively mobilized, demonstrating the role of viscosity enhancement in improving oil recovery.

**FIGURE 12:**
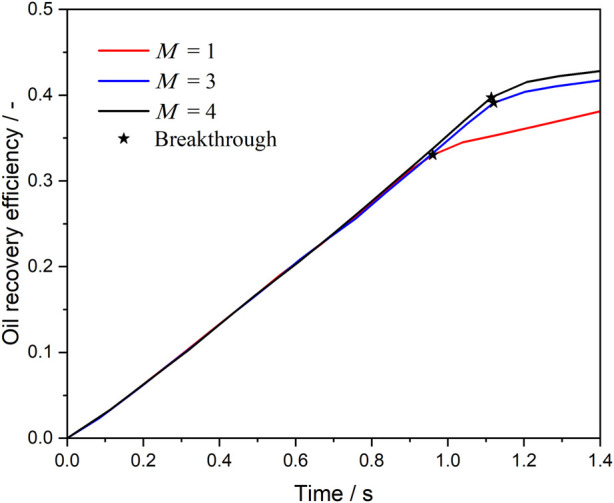
Oil recovery efficiency during microemulsion-forming surfactant flooding at different viscosity ratio.

Figure 12 presents the oil recovery efficiency in the complex pore channel under different viscosity conditions, with a fixed oil-water-rock contact angle of 30°. When no viscosity enhancement is applied (*M* = 1), the oil saturation in the pore space is the highest, indicating low recovery efficiency. However, when the viscosity of the *in situ*-formed microemulsion is increased by a factor of 3 (*M* = 3), the oil saturation decreases significantly by approximately 8%, highlighting the substantial improvement in recovery due to viscosity enhancement. Interestingly, further increasing the viscosity ratio to *M* = 4 yields a similar recovery improvement compared to *M* = 3, suggesting diminishing returns with higher viscosity ratios. This observation provides valuable insights for the development of chemical agents: while increasing the viscosity of *in situ*-formed microemulsions enhances oil recovery, the relationship is not linear. Therefore, a balance must be struck between field requirements and economic costs to achieve significant recovery improvements at lower operational expenses.

### 3.4 Effect of wettability on microemulsion-forming surfactant flooding

**FIGURE 13:**
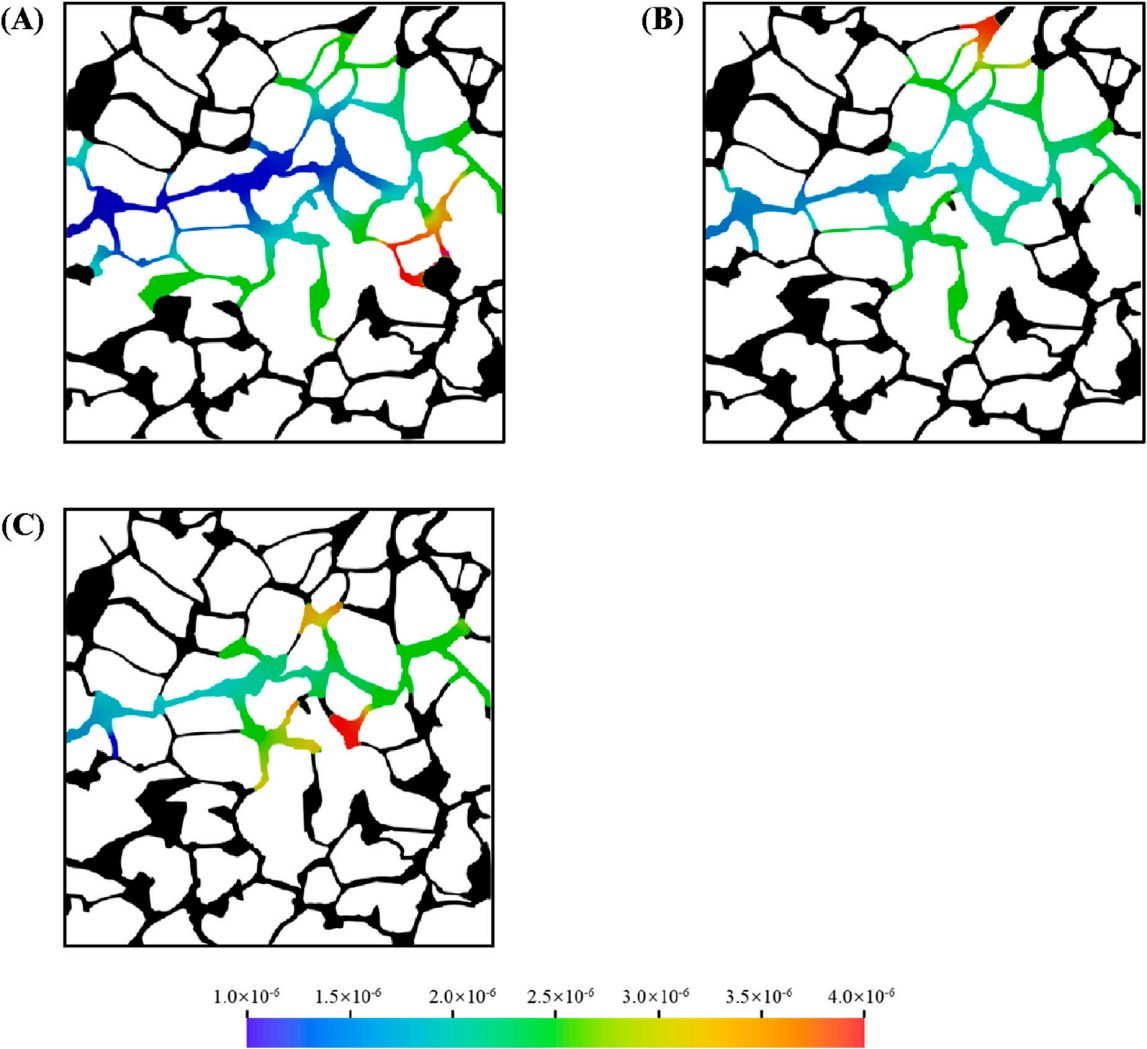
Oil and aqueous distribution in porous media during microemulsion-forming surfactant flooding at different: **(A)** 30°, **(B)** 90° and **(C)** 150°.

Wettability in oil reservoirs refers to the tendency of water to preferentially contact the rock surface in the presence of oil. Solid surfaces can range from strongly water-wet to strongly oil-wet, depending on mineral composition and the thermophysical properties of the fluids. Wettability significantly influences multiphase flow in porous media and is typically characterized by the contact angle. To investigate the effect of wettability on the *in situ* formation in complex structure, three contact angles were simulated: 30°, 90°, and 150°. Figure 13 shows the oil and water distributions in the porous medium with different contact angle. The color represents the concentration of *in situ* formed microemulsion in water during microemulsion-forming surfactant flooding. The oil-water interface stops at the junction from the throat to the pore under water-wet and neutral wetting conditions (Figures 13A,B). However, under oil-wet conditions, the situation is reversed and the oil-water interface always stays at the junction from the pore to the throat (Figure 13C). This phenomenon can be explained as capillary barrier, which has been analyzed detailly in our previous studies. In addition, wettability significantly affects the formation of mainstream channels during two-phase displacement. The dominant channel within the porous medium is more pronounced under water-wet conditions.

**FIGURE 14:**
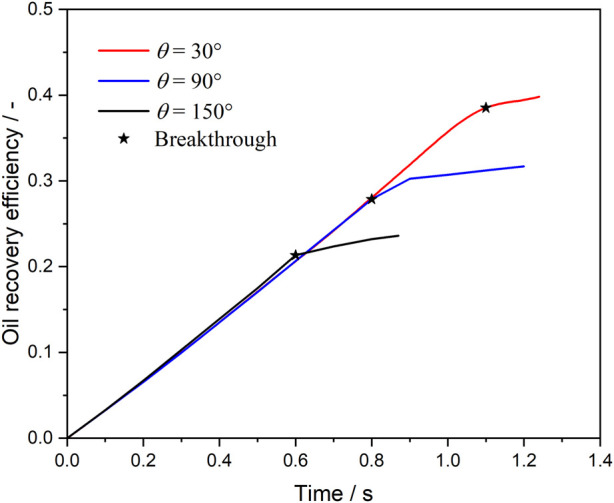
Oil recovery efficiency during microemulsion-forming surfactant flooding at different contact angles.

Figure 14 shows the oil recovery efficiency during microemulsion-forming surfactant flooding in the complex porous media with different contact angles. Before the injection time of 0.6 s, the decreasing trend of oil saturation under different wetting angle conditions is basically the same, but then there will be a significant difference. For the oil-wet condition, the decrease of oil saturation becomes slower after 0.6 s at a wetting angle of 150°. For strong water-wet conditions, on the other hand, at a wetting angle of 30°, the oil saturation continues to decrease after 0.6 s and does not turn around until after 1.1 s. The variability of the microemulsion replacement process under different wettability conditions can be observed more intuitively in Figure 14. Although the distribution of mainstream channels is the same under the three wettability conditions, the wave area of microemulsion drive is significantly wider under strong water-wet conditions.

## 4 Conclusion

In this study, a novel pore-scale numerical simulation model was proposed to capture the *in situ* microemulsion formation during surfactant solution flooding in porous media. Then, a series of numerical simulation were conducted to explore the dynamics distribution of oil and *in situ* formed microemulsion during microemulsion-forming surfactant flooding in a real rock medium. The influence of the wettability and viscosity of microemulsion on the enhanced oil recovery was analyzed. The primary conclusions are summarized as follows:

1. The proposed novel pore-scale numerical simulation model is able to simulate the dynamic formation process of oil-in-water microemulsions within the pore space. By considering some typical characteristics of *in situ* formed microemulsions, including increased viscosity, reduced interfacial tension, altered wettability, and solubilization, it is analyzed how these characteristics enhance recovery.
2. During the microemulsion-forming surfactant flooding in a real rock medium, the *in situ* formed microemulsion was observed at the interface between water and aqueous. The *in situ* microemulsion flooding can significantly improve the recovery rate under the combined effect of multiple factors. For example, the solubilization effect was able to gradually dissolve the residual oil droplets in the pores, while the wettability change was able to reduce the formation of the residual oil droplets in the pores
3. Increasing the viscosity of the *in situ* formed microemulsion can enhance the oil recovery during the microemulsion-forming surfactant flooding in the complex porous media. However, the viscosity of the microemulsion is not linearly related to the recovery rate, and in real mines it is necessary to consider the economic cost to choose the microemulsion viscosity increasing.
4. Wettability can significantly affect the oil-water two-phase displacement process within porous media, which further affects the *in situ* formation process of microemulsions. Under water-wet conditions, the oil-water interface stays at the junction of the throat and the pore space, which contributes to the formation of microemulsions and thus to the enhancement of recovery.

It is worth noting that the proposed model is based on the Navier-Stokes equations, which fundamentally restricts the analysis to conventional fluid dynamics. Consequently, the framework may not accurately capture flow behaviors in unconventional reservoirs (e.g., shale formations) where nanoscale pore structures dominate the transport phenomena. In addition, while this investigation provides valuable insights into oil-water displacement mechanisms at the pore scale and identifies key factors governing microemulsion-driven recovery enhancement, direct field application remains challenging due to the inherent scale differences between laboratory investigations and reservoir conditions.

## Data Availability

The original contributions presented in the study are included in the article/supplementary material, further inquiries can be directed to the corresponding author.
